# Anæsthetics—Use and Results

**Published:** 1883-07

**Authors:** 


### SELECTIONS.

### Anæsthetics—Use and Results.

[Before Marion Co., Ind., Medical Society.]

BY DR. FERGUSON.

There are a great number of volatile substances, which, when inhaled, are capable of producing anæsthesia, the majority of which are either alcoholic or of alcoholic origin, and their physiological effects resemble each other so closely that a description of the action of ether or chloroform conveys a general idea of the phenomena produced by all the agents of this class.

After briefly describing the effects produced by local contact of anæsthetics, Dr. Ferguson said:

The first systemic effect is disturbance of function; the next, paralysis of function. This disturbance of function generally assumes the form of exaltation. The special senses are the first to give evidence of this disturbance; the patient is conscious of a humming sound in the ears, and subjective impressions of light flash across the visual field. General sensibility is also exalted. The patient is conscious of the beatings of his own heart, and can feel the vermicular movement of his intestines ; the arteries throb, the brain is excited, respiration accelerated, pulse rises, pupils contract, eyes close, and reflex irritability is exalted. If the inhalation be continued, this general excitement is soon succeeded by the stage of diminished function, lessened sensibility of the surface, and fall of temperature ; pulse, which had risen during the stage of excitement to 100 or more, now falls toward the normal standard ; the blood pressure diminishes ; respirations, which had risen during the period of excitement, fall below the normal standard, and become deep and full, resembling those of normal sleep ; consciousness fails, voluntary movements cease, reflex movements are abolished, the wild ravings of the patient gradually subside, he becomes insensible, the power of voluntary motion is abolished, and the only movements which persist are those which sustain the functions of respiration, circulation, and vegetative life.

How are the effects of anæsthetics produced ? It was thought by early experimenters that the insensibility produced by anæsthetics was the result of mechanical compression of the brain. According to their opinions, the vapors of these substances possessed such a degree of "tension" that, when the blood was charged with them, it produced compression of the brain, analogous to that which takes place after effusions into the cranial cavity. But it is now known that they do not exist in a vaporous condition within the body, but are dissolved in the blood, and, of course, can produce no more pressure than is produced by the current of blood itself. Others have believed that the anæsthetic state is produced by asphyxia. The carbon, a large proportion of which exists in the majority of anæsthetic substances, is supposed to deprive the blood of its oxygen, thus producing an asphyxiated condition of the nervous tissues. But it is known that the chemical composition of the blood is not altered in a way that is consistent with this theory. The blackness which is characteristic of the blood in asphyxia is not present in anæsthesia, but is of a dark brown color, and its corpuscles may, or may not, present any change in shape or condition.

It is probable that the essential cause of the phenomena of anæsthesia is molecular paralysis. Dr. Henry M. Lyman, of Chicago, has shown that "the action of vegetable protoplasm upon carbonic acid gas, by virtne of which plants are enabled to absorb carbonic acid gas, and to exhale oxygen when exposed to the rays of light, is arrested by the presence of anæsthetic substances in the fluids of the plant." By immersing a healthy specimen of an aquatic plant in a watery solution of ether or chloroform, and inverting a bell-glass over the vessel in which it is placed, he found that the absorption of carbonic acid gas, and the exhalation of oxygen ceased, then removing it and placing it in pure water, the processes of vegetable respiration were renewed.

Claude Bernard has shown that the germination of seeds is arrested by the action of anæsthetics. He surrounded seeds with the vapor of ether which had otherwise been placed in conditions suitable for germination. At the expiration of six days no change had taken place; pure air was then substituted for the vapor of ether and the seeds at once began to germinate. He also proved by experiment, that the irritability of the protoplasm found in the cells at the base of the petiole in the sensitive plant was suspended by the presence of the vapor of ether, and after the ether was removed, the effects gradually passed off and the irritability of the plant returned. The microscopic cellular element of which the animal frame is composed are similar to those of which the plant is constructed. So far as microscopic appearances are concerned, animal and vegetable protoplasm, or bioplasm, as you may please to call it, are identical, the only difference being, that in animals there is a greater degree of differentiation, among the individual elements; for instance, there are certain groups of elements which are constructed for the special work of secretion, others are specially constructed for the purpose of contractility, others for the reception of sensitive impressions, and still others for the initiation of motion. These differences imply great variation of structure, and we might *a priori* infer that some of them would be much more easily affected by anæsthetic agents than others. Practically we find this to be the case, certain cell groups yielding quickly to the paralyzing influence of an anæsthetic agent, while others manifest considereble resistance. This fact explains the progressive character of the phenomena of anæsthesia.

The elementary structures of the nervous system are the first to yield to the influence of anæsthetics, and the sensory apparatus succumbs to their influence sooner than the motor. This fact is illustrated by an observation of Claude Bernard, who found that the muscles could be roused to action in a chloroformed frog, by irritating the sciatic nerve, when no evidence of sensation could be made apparent. Certain portions of the nervous apparatus yield sooner than others. The muscular paralysis of anæsthesia advances from below upward, and from the periphery toward the center, the medullary centers being the last to become paralyzed. As the power of liberating molecular motion is most completely organized in the medulla oblongata, and as the functions of respiration and circulation are controlled by this center, they persist after all other motor functions in other portions of the nervous system have ceased.

Having said this much upon anæsthetics in general, and as ether and chloroform are the two agents in general use, and in view of the fact that many deaths have occurred under their use, the question arises, what are the dangers in their use and which is the safer agent? In reviewing the histories of patients who have died from the effects of ether and chloroform, we find that death may occur at any stage of the inhalation. It may occur suddenly at the commencement of inhalation, without any preliminary excitement, the patient expiring as suddenly as if a powerful current of electricity had been passed through the medulla. It may occur in the stage of excitement, the muscular system becoming suddenly tetanized, producing fatal results by asphyxia. It may occur after the patient has been completely anæsthetized, or even after the effects of the agent have passed off and the patient recovered consciousness. In general, it may be said that death occurs in one of the following ways:

1. Shock.
2. Asphyxia.

3. Failure of the heart's action.
4. Failure of the respiration.
5. Failure of both circulation and respiration.

Death from shock occurs at the commencement of the inhalation, and is probably allied to those cases of sudden death which occur from the shock produced by sudden emotions of fright, joy, etc. Death from asphyxia may occur either from spasmodic condition of the general muscular system, or from spasm of the glottis alone, or from a complete saturation of the tissues by the stupefying agent, preventing the normal interchange of the carbonic acid and oxygen in the tissues of the body. But the most frequent cause of death is from the failure of the circulation and respiration, and it is to these that the attention of the physician must always be directed when administering anæsthetics. Which is the safer agent, ether or chloroform? Paul Bert's recent researches have shown whenever a certain relative quantity of any anæsthetic has been introduced into the blood asphyxia is always the result. Lesser degrees of impregnation produce anæsthesia alone. In experiments upon the dog, the animal had to breathe an atmosphere charged with the vapor of ether in proportion to 37 grammes of ether to 100 litres of air in order to produce anæsthesia. If the quantity of ether was doubled, the animal died of asphyxia. In order to produce anæsthesia with chloroform it required only 15 grammes to 100 (61.027 cubic inches) litres of air, and when the quantity was increased to 30 grammes asphyxia and death were the result. It is an easy thing to impregnate 100 litres of air with 30 grammes of chloroform, but a difficult matter to impregnate the same amount of air with 74 grammes of ether, hence the danger from asphyxia by chloroform is far greater than from ether.

Is there more danger of paralysis of the respiratory and circulatory centers by chloroform than by ether? It was formerly taught that the action of these two agents upon the heart was different—that ether stimulated the heart's action, and chloroform depressed its action; that ether produced paralysis of the lungs, and chloroform paralysis of the heart. But it is now known by direct experiment that the effects of both upon the respiration and circulation are similar, differing only in intensity. Both stimulate the heart and respiration at the commencement of the inhalation; both depress the action of the heart and respirations, as their secondary effects, ether producing more stimulation and less depression, chloroform producing less stimulation and more depression.

When either of these agents are inhaled they pass directly through the walls of the capillaries and become dissolved in the blood in the lungs; they are then carried to the left side of the heart, forced into every portion of the organ through the coronary arteries, and through the aorta and its divisions are carried throughout the entire system. The first effects are experienced by the tissues which constitute the walls of the pulmonary veins and coronary arteries. The ganglia of their vaso-motor nerves are placed in these coats, and upon these the paralyzing effects of the anæsthetic is directed as well as upon the other tissues of the cardiac blood-vessels. After a brief period of contraction, the arterioles relax, and as the result of this relaxation a larger supply of blood is distributed to the muscular tissues of the heart and its ganglia, and this increases the function of both its nerves and muscle; the heart's contractions increase in vigor, and more blood is sent to the brain and every portion of the body, and this arouses the activity of the nervous tissue everywhere. But this excitement is of short duration, the blood becoming more thoroughly charged with the anæsthetic, its paralyzing influence invades the higher nervous ganglia, and as the functional activity of these ganglia is lessened, their stimulating influence on the circulation is diminished, and the pulse rate falls toward the normal standard. If now an equilibrium between the introduction and elimination of the agent is established, anæsthesia will be continuously sustained, and with the same degree of intensity, so long as this equilibrium be maintained; but if the vapor is introduced faster than it can be eliminated, the tissues soon become supersaturated and death is the final result. Death, of course, will be more likely to follow when an energetic and concentrated agent is used, and for this reason chloroform is a more dangerous anæsthetic than ether.

The doctor then presented statistics showing the greater fatality attending the use of chloroform as an anæsthetic over ether; also the experiments of the committee of the British Medical Association, proving that asphyxia was more readily produced in frogs by chloroform than by ether, etc., and closed by the observation that with our present knowledge of the action of these two agents, and the greater safety of ether, that the indiscriminate use of chloroform was not justifiable, and that in medico-legal investigations of cases of death occurring from its use as an anæsthetic, the physician must be able to show that there was no existing contra-indications to its use, and that every precaution was taken to avoid accidents. In presenting this subject, I lay no claim to the advancement of anything new, but have freely availed myself of standard authors, and more especially of the work of Dr. Lyman, of Chicago.—*Indiana Medical Journal.*

